# UV-Induced Nuclear Import of XPA Is Mediated by Importin-α4 in An ATR-Dependent Manner

**DOI:** 10.1371/journal.pone.0068297

**Published:** 2013-07-08

**Authors:** Zhengke Li, Phillip R. Musich, Brian M. Cartwright, Hui Wang, Yue Zou

**Affiliations:** Department of Biomedical Sciences, East Tennessee State University, J.H. Quillen College of Medicine, Johnson City, Tennessee, United States of America; University of Medicine and Dentistry of New Jersey, United States of America

## Abstract

Xeroderma pigmentosum Group A (XPA) is a crucial factor in mammalian nucleotide excision repair (NER) and nuclear import of XPA from the cytoplasm for NER is regulated in cellular DNA damage responses in S-phase. In this study, experiments were carried out to determine the transport mechanisms that are responsible for the UV (ultraviolet)-induced nuclear import of XPA. We found that, in addition to the nuclear localization signal (NLS) of XPA, importin-α4 or/and importin-α7 are required for the XPA nuclear import. Further investigation indicated that, importin-α4 and importin-α7 directly interacted with XPA in cells. Interestingly, the binding of importin-α4 to XPA was dependent on UV-irradiation, while the binding of importin-α7 was not, suggesting a role for importin-α7 in nuclear translocation of XPA in the absence of DNA damage, perhaps with specificity to certain non-S-phases of the cell-cycle. Consistent with the previous report of a dependence of UV-induced XPA nuclear import on ataxia telangiectasia and Rad3-related protein (ATR) in S-phase, knockdown of ATR reduced the amount of XPA interacting with importin-α4. In contrast, the GTPase XPA binding protein 1 (XAB1), previously proposed to be required for XPA nuclear import, showed no effect on the nuclear import of XPA in our siRNA knockdown analysis. In conclusion, our results suggest that upon DNA damage transport adaptor importin-α4 imports XPA into the nucleus in an ATR-dependent manner, while XAB1 has no role in this process. In addition, these findings reveal a potential new therapeutic target for the sensitization of cancer cells to chemotherapy.

## Introduction

Human genomic DNA is constantly exposed to endogenous and exogenous damaging agents, which may lead to genome instability. Removal of these structural and chemical abnormalities in DNA requires timely and coordinate recruitment of DNA repair factors to the damaged DNA [1], [2]. The NER pathway is the primary mechanism in cells for removal of helix-distorting, replication-blocking bulky DNA adducts that are induced by exogenous agents such as UV radiation and a variety of genotoxic chemicals [1], [2]. In humans, defects of NER lead to the clinical disorder *xeroderma pigmentosum* (XP) which is characterized by an increased sensitivity to UV radiation and a predisposition to development of skin cancers [3], [4]. The XPA protein is one of eight factors that were found to be deficient in XP disorders [5], and the XPA-deficient cells exhibit the highest UV sensitivity among the XP cells [6]. XPA is an indispensable factor both for transcription-coupled NER and global genome NER [7], [8]. Functionally, XPA is believed to play roles in verifying DNA damage, stabilizing repair intermediates, and recruiting other NER factors to the damaged DNA [1], [9], [10], [11], [12], [13], [14], [15], [16], [17], [18]. Because of XPA’s crucial functions in NER, the activity of NER and sensitization of cancer cells to chemotherapy can be regulated by transcriptional and post-transcriptional control of the XPA protein [19], [20], [21], [22], [23], [24], [25], [26]. In addition, recent studies demonstrated that besides its anticipated functions in NER, XPA also may participate in other cellular events in the absence of genotoxic insults, such as facilitating chromatin modification for transcription [27], inhibiting DNA double-strand break repair in progeria [28], and potential involvement in DNA replication [29].

XPA is essential for the NER process which occurs in the nucleus, consistent with the initial description of XPA as a nuclear protein [30]. However, more recent biochemical fractionation and immunofluorescence studies demonstrate that in unstressed cells XPA is primarily restricted to the cytoplasm of cells in G_1_ and S phases, though it is present predominately in the nucleus during the G_2_ phase [24], [25], [31], [32]. Given the important nuclear functions of XPA in NER [1], [31], it is of interest to study the DNA damage-dependent nuclear import of XPA [24], [32], [33], and also the potential to target protein trafficking as a strategy to improve the sensitivity of cancer cells to chemotherapy agents [34]. It was shown previously that the DNA damage-induced nuclear import of cytoplasmic XPA for NER during S-phase is a DNA damage checkpoint-dependent process mediated by ataxia telangiectasia and Rad3-related (ATR) protein and p53 tumor suppressor protein [24], [25], [33]. Both ATR and free p53 localize in the nucleus with or without DNA damage. However, it remains unresolved how XPA is imported into the nucleus from the cytoplasm. Also, little has been done to understand the mechanisms for potential regulation of the nuclear import of any DNA repair proteins.

In this study, protein factors potentially involved in the transport of XPA through the nuclear pore complex (NPC) were investigated. We found that in the presence of the nuclear localization signal (NLS) of XPA, importin-α4 and importin-α7 served as the transport adaptors for the UV-induced nuclear import of XPA from the cytoplasm. Consistent with the requirement of ATR for the UV-induced XPA nuclear import, the role of importin-α4 in the import also was dependent on ATR. Surprisingly, however, XAB1 protein, suggested previously to be the GTPase involved in XPA nuclear import, showed no effect on the XPA nuclear import. Given the indispensable role of XPA in human NER, our findings demonstrate a cytoplasmic regulatory mechanism important for NER. The therapeutic disruption of this transport process may provide a means of sensitizing cancer cells to chemotherapeutic drugs.

## Materials and Methods

### Cell Culture, UV-irradiation and Antibodies

*Xeroderma pigmentosum* fibroblast cells GM04429 (XPA^−/−^) was purchased from Coriell. A549, HEK 293T, H1299, and BJ cells were from American Type Culture Collection. The human cell lines used in these studies were grown in D-MEM supplemented with 10% FBS and 1% penicillin-streptomycin at 37°C, 5% CO_2_. The UV-induced translocation from cytoplasm to nucleus was clearest in H1299 cells though it has been observed in multiple human cancer, transformed and primary cells lines ([26], [32], [33] and data not shown). Thus, H1299 cells were used for most of the studies reported here. A549 cells were used to study the ATR-dependence of the XPA-importin interactions since this is a p53-dependent phenomena [32]. The GM04429 cells were essential in the experiments that required cell extracts completely free of endogenous XPA since they are naturally XPA deficient.

XPA-complemented cells were generated by stably transfecting H1299 cells with pcDNA3.1 vectors (Invitrogen) containing either wild type or NLS-mutated XPA cDNA with the mutations indicated in the text. UV-C irradiation was performed using a 254 nm lamp at a flounce of 0.83 J/m^2^/sec. For probing western blots primary rabbit polyclonal antibody against XPA and mouse monoclonal antibody against poly (ADP-ribose) polymerase (PARP) were purchased from Santa Cruz Biotechnology Co. Antibodies against importins-α1, -α3, -α4, -α5, or -α7 were from Genetex Biotechnology Co. A FITC-conjugated primary mouse anti-actin antibody was obtained from Sigma Chemical Co. The anti-actin and anti-PARP antibodies were used in western blots to confirm successful subcellular fractionations and protein loadings. The rabbit monoclonal antibody against XAB1 is from Sigma Chemical Co.

### Immunoblotting

Cells were harvested by scraping or trypsin-EDTA release, and re-suspended in lysis buffer [50 mM Tris-HCl, pH 7.8, 150 mM NaCl, 1 mM EDTA, 1% Triton X-100, 1× protease inhibitor cocktail (Roche)]. 2× SDS loading buffer was added to the lysates and the mixtures were heated at 100°C for 10 min to denature proteins. After running the samples in SDS-PAGE, proteins were transferred onto a PVDF membrane. The membrane then was blocked with 5% nonfat dry milk in TBST buffer and probed with specific primary and secondary antibodies. Chemiluminescence signal was captured using a Fuji Film camera, and the blot images were processed with Multi-Gauge 3.0 software.

### RNA Interference (RNAi)

XAB1 siRNA duplexes were purchased from Santa Cruz Biotechnology Co. siRNA duplexes targeting individual importin-α proteins in lung cells were successfully developed by Gabriel *et al.* [35]. These sequences were synthesized by Genepharam. The siRNA transfection reagent was purchased from Polyplus Transfection and the transfections were done by following their instructions as we described previously [32], [33].

### Subcellular Fractionation

Subcellular fractionation was performed using the Proteo JET™ cytoplasmic and nuclear protein extraction kit (Fermentas) by following the procedures suggested by the manufacturer. Briefly, 10 volumes of cell lysis buffer (with 1× protease inhibitors) were added to 1 volume of packed cells. After a short vortexing and incubation on ice for 10 min, cytoplasm was separated from nuclei by centrifugation at 500×g for 7 min at 4°C. Isolated nuclei were washed once or twice with 500 µL of the nuclear washing buffer and then collected by centrifugation. The collected nuclear pellets were re-suspended in ice-cold nuclear storage buffer, and 1/10 volume of the nuclear lysis reagent was added to lyse the nuclei with rotation for 15 min at 4°C. A clarified nuclear lysate was obtained by centrifugation at 20,000×g for 15 min at 4°C. In all of the fractionation experiments western blotting of β-actin and PARP were assessed to check successful fractionation and cytoplasmic and nuclear protein loading, respectively.

### Immunoprecipitations

Cells were lysed with NETN lysis buffer (20 mM Tris-HCl [pH 8.0], 100 mM NaCl, 1 mM EDTA, 0.5% Nonidet P-40) containing protease and phosphatase inhibitors (Thermo Scientific). For each 2 mg of protein in the whole cell lysates 2 µg of primary antibody was added and the mixture incubated at 4°C with agitation overnight. Then, 15 µl of high-capacity protein G-agarose (Thermo Scientific) was added and incubated for 2 hr at 4°C to capture the antibodies. After washing three times with NETN buffer, proteins were released from beads in SDS sample loading buffer with heat denaturation, resolved on 10% SDS-PAGE, and subjected to western blotting. Immunoprecipitation of XPA-binding proteins was conducted by mixing 1.0 µg of purified recombinant XPA protein with 2 mg of cell lysates from mock or UV-irradiated GM04429 (XPA^−/−^) cells; a goat monoclonal antibody to the C-terminal of XPA (Santa Cruz) then was added to the lysates and incubated at 4°C with agitation overnight. Protein G-agarose beads were added to capture the antibodies, the beads were washed 3 times with NETN buffer, and bound proteins eluted with SDS sample buffer for analysis on 10% SDS-PAGE, and subjected to western blotting.

### *In vitro* Protein-protein Interaction

To characterize importin-XPA interactions *in vitro* cellular importin proteins were first isolated by immunoprecipitation with importin-α4 or importin-α7 specific antibodies. To remove any co-precipitated endogenous XPA protein the immunoprecipitated importin proteins were washed for 5 minutes at 4°C with a high salt buffer (25 mM Tris-HCl [pH 8.0], 0.6 M NaCl, 0.2% NP-40, 1 mM EDTA) containing protease inhibitors (Thermo Scientific). This high salt buffer was shown previously to remove associated proteins from immunoprecipitates [25], [36]. After collection by centrifugation the beads and bound proteins were re-suspended in 200 µl of NETN buffer. Then, 0.5 ug of purified XPA protein was added to each sample and incubated overnight at 4°C with agitation. The beads with associated proteins were collected by centrifugation and washed three times with NETN buffer, released in SDS sample buffer, resolved on 10% SDS-PAGE and subjected to western blotting.

### Immunofluorescence Microscopy

Cells were grown on coverslips before the initiation of experimental treatments. After UV-C irradiation and specified recovering times, the cells were fixed with 100% cold methanol and blocked with 15% BSA for 1 hr at room temperature. Proteins were detected with primary antibodies and fluorescence-conjugated secondary antibodies (Invitrogen). Cells on coverslips were coated with prolong gold anti-fade reagent containing DAPI (Invitrogen) before microscopic examination using 100× magnification.

## Results

### The N-terminal NLS of XPA is Required for UV-induced Nuclear Import

**Figure 1:**
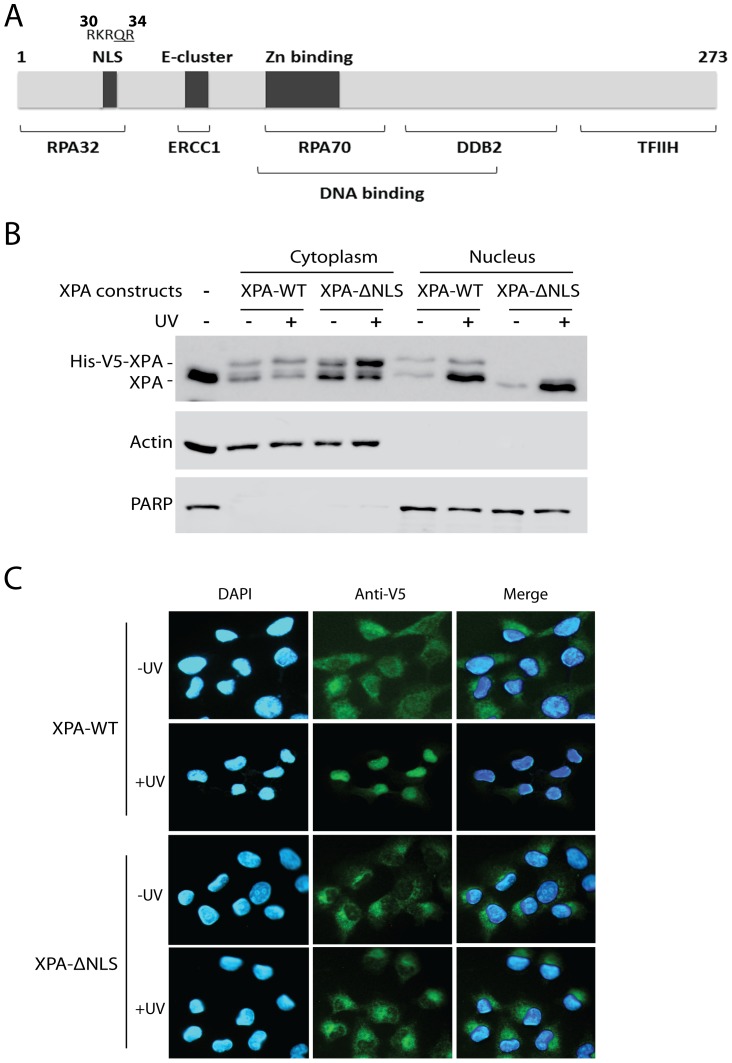
Nuclear localization of recombinant XPA requires an N-terminal NLS sequence. **A.** A map of XPA protein illustrating the locations of the binding sites for various DDR proteins or for binding damaged DNA [37]. The numbers refer to the first and last amino acid in the XPA protein or the residues #30-#34 of the NLS sequence. The XPA-ΔNLS protein construct was made by changing the amino acids Q33 and R34 (underlined) within the NLS to alanine by PCR mutagenesis. **B.** Subcellular fractionation and Western blotting demonstrate UV-induced and NLS-dependent XPA redistribution from the cytoplasm to the nucleus. Stably transfected H1299 cells were mock or UV-C irradiated (20 J/m^2^) followed by a 2-hr recovery. The recombinant N-terminal 6xHis-V5-tagged XPA protein migrates slower than endogenous XPA allowing us to detect each XPA protein using XPA antibody. **C.** Immunofluorescence microscopy of recombinant XPA using antibody against the V5-tag portion of the recombinant XPA protein. H1299 cells were treated as in A. The localization of XPA was assessed by immunofluorescence microscopy. The nuclei were stained with DAPI.

To maintain a functional NER, XPA protein contains multiple functional domains for interaction with various other proteins, including RPA (replication protein A), excision repair cross-complementation group 1 (ERCC1), DNA damage-binding protein 2 (DDB2), and transcription factor II H (TFIIH), as well as chemical carcinogen-damaged or UV-damaged DNA [37] (Figure 1A). Among these domains, a NLS-like motif -RKRQR-) is located in the N-terminus (residues 30–34) [38] (Figure 1A). To determine whether the DNA damage-induced nuclear import of XPA depends on this NLS-like motif or whether XPA is co-imported with proteins containing a NLS, or imported by the so-called “alternative import mechanisms” [31], two last amino acids of the XPA NLS [38] were replaced with alanine (XPA-ΔNLS). As shown in Figure 1B and Figures S1A–Figures S1C, no mutated XPA protein (His-V5-XPA) was detected in the nuclear fractions of these mutated transfectants even after UV-irradiation. In comparison, after UV-irradiation the endogenous XPA increased in the nucleus as it decreased in the cytoplasm, and the recombinant wild-type XPA also accumulated in the nucleus (Figures 1B, S1A–S1C), indicating a UV-induced nuclear import of wild type recombinant XPA. Similarly, the immunofluorescence microscopy analysis showed that XPA-ΔNLS remained in the cytoplasm even after the cells were irradiated with UV, while the wild-type XPA accumulated normally in the nucleus (Figure 1C). These results suggest a dependence of the DNA damage-induced XPA nuclear import on the protein’s NLS motif. It should be noted that as compared to the endogenous XPA, the level of His-V5-XPA expressed from transfected construct appeared not being affected by UV in the cytoplasm as analyzed by the fractionation assay (Figure 1B). It is possible that expression of the transfected XPA construct might be stimulated to compensate the loss of XPA in the cytoplasm due to the UV-induced nuclear import. This could be true since the transient transfection was likely to have most of the transfected constructs in the cytoplasm rather than in the nucleus.

### Importin-α4 and Importin-α7 are Involved in the UV-induced XPA Nuclear Import

**Figure 2:**
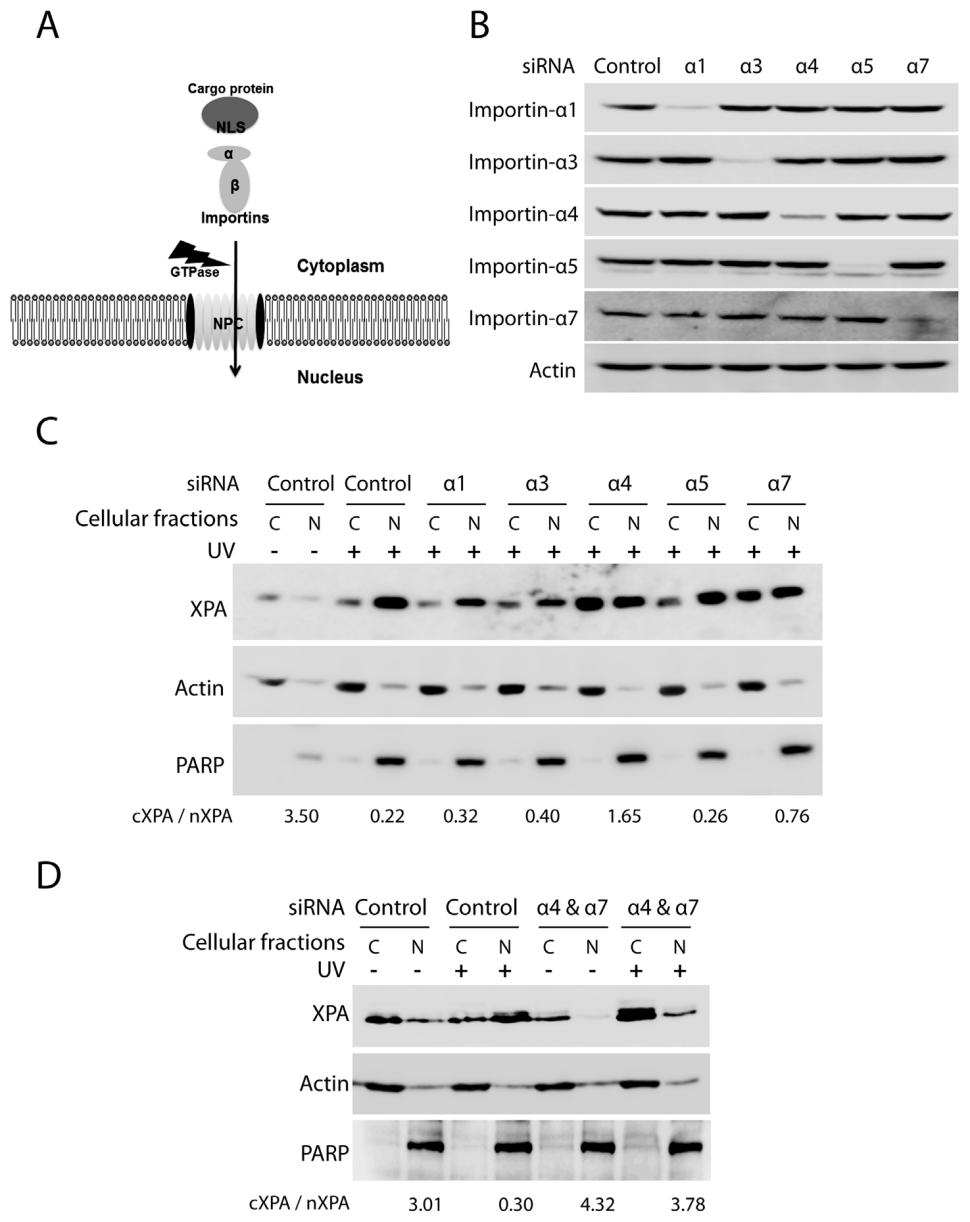
Importin-α4 and -α7 are involved in the nuclear import of XPA. **A.** A model for the importin-α and –β facilitated movement of proteins into the nucleus. **B.** Western blotting confirms the efficiency of siRNA knockdown of each of the five importin-α proteins. H1299 cells were transiently transfected with siRNAs targeting individual importin-α proteins. Whole cell lysates were prepared 48 hours post-transfection and assessed by western blotting. **C.** Fractionation and western blotting assess the localization of XPA following UV-irradiation. H1299 cells were transfected with the indicated siRNA duplexes and, at 48 hours post-transfection, cells were mock or UV irradiated (20 J/m^2^) and allowed a 2-hr recovery. Cytoplasmic and nuclear lysates were collected and analyzed by western blotting of XPA. The numbers below the figure represent the ratio of the amount of cytoplasmic XPA divided by the amount of nuclear XPA (cXPA/nXPA) and were calculated from repeated experiments. **D.** Similar experiments as in panel C except that importin-α4 and importin-α7 were silenced simultaneously.

XPA has an N-terminal NLS (Figure 1), but its localization to the nucleus where it functions also depends on DNA damage and cell cycle stage [24], [32], [33]. These observations indicate that there maybe two stages of XPA nuclear import: one occurs naturally in the absence of DNA damage, and another, the massive transport (particularly in S-phase), is initiated by specific DNA damage signaling [24], [32], [33]. Among these factors involved in the DNA damage-induced XPA import, those involved in the XPA transport through the NPC are unknown. Importin-α is a constituent of the classical nuclear import pathway. It acts as an adaptor protein that recognizes the NLS of cargo proteins and is transported as a ternary complex with importin-β into the nucleus [39], [40] (Figure 2A). In humans, six importin-α isoforms are known [41], [42], [43], [44]. To identify the importin-α responsible for the UV-induced nuclear import of XPA, importin-α proteins in human cells were silenced by siRNA knockdown (Figure 2B). Subsequent subcellular fractionation analysis indicated that knockdown of importin-α4 or/and importin-α7 inhibited the XPA nuclear import (Figure 2C and 2D): XPA was present primarily in the cytoplasm without UV-irradiation in control cells, and was transported into the nucleus after UV-irradiation in the control and importins-α1, -α3 or -α5 siRNA-knocked down cells (as indicated by the low ratio of cXPA/nXPA). However, in cells with importin-α4 and/or importin-α7 silenced with siRNAs, a significant amount of XPA remained in the cytoplasm.

The similar experiments were also carried out in the cells without UV treatment. As shown in Figure S1D, without UV the siRNA-knockdown of importin α4 had no effect on XPA subcellular localization, indicating that the knockdown effect of importin α4 is UV-dependent. In contrast, knockdown of importin α7 showed some effect on XPA subcellular localization in the absence of UV, indicating that the knockdown effect of importin α7 is UV-independent.

### Interaction of Importin-α4 and Importin-α7 with XPA

**Figure 3:**
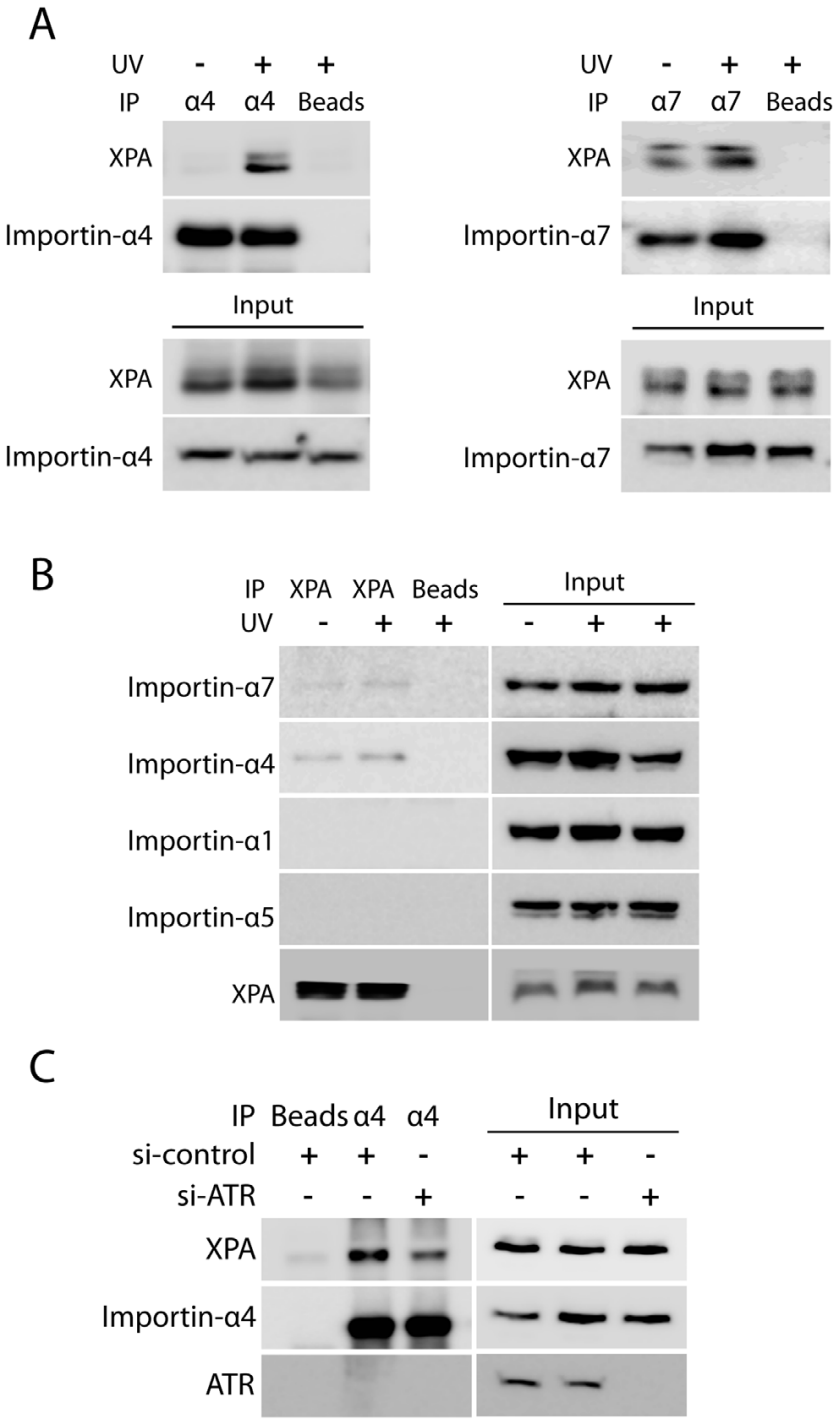
Importin-α4 and importin-α7 form complexes with XPA in cells. **A.** XPA was detected in the protein complexes of importin-α4 or importin-α7 by Western blotting. Immunoprecipitation of importin-α4 or importin-α7 also pulled down XPA in lysates from H1299 cells which were UV-C irradiated (20 J/m^2^) and allowed a ½ h recovery compared with lysates from cells that were mock treated. **B.** A reciprocal experiment in which XPA was immunoprecipitated using a C-terminal XPA antibody. GM04429 (XPA^−/−^) cells were mock or UV-C irradiated (20 J/m^2^) followed by a ½ h recovery. Cell lysates were prepared and supplied with recombinant XPA protein. Immunoprecipitation of recombinant XPA was employed to assess whether the added XPA formed complexes with importin-α4 or importin-α7 proteins. **C.** Knockdown of ATR inhibited cellular interaction of XPA and importin-α4. A549 cells were transfected with control (scrambled) or ATR siRNA. 72 hours post-transfection cells were mock treated or exposed to UV-C radiation (20 J/m^2^), followed by a 1/2-hr recovery. The amount of XPA associated with the immunoprecipitated importin-α4 was assessed by Western probing of XPA.

Cargo protein with a classic NLS requires a direct interaction with importin-α to be transported through the nuclear pore complex [31], [40], [45], [46], [47] (Figure 2A). Further experiments were carried out to investigate if XPA forms complexes with importin-α4 or importin-α7. As shown in Figure 3A, XPA was co-immunoprecipitated with importin-α4 or importin-α7 antibodies. As a comparison, incubation of the cell lysates with protein G-agarose did not pull down any detectable XPA; suggesting that the presence of XPA in the immunoprecipitates is due to the interaction of importins-α4 or -α7 with XPA. Interestingly, while little XPA was found to bind to importin-α4 in cells prior to UV treatment, XPA was co-precipitated with importin-α4 after ½ hr of recovering from 20 J/m^2^ of UV-irradiation (Figure 3A, left panel), indicating the involvement of importin-α4 in the UV-induced XPA nuclear import. In contrast, XPA was co-precipitated with importin-α7 even in the absence of UV treatment (Figure 3A, right panel). These results were well consistent with those shown in Figures 2C and S1D.

**Figure 4:**
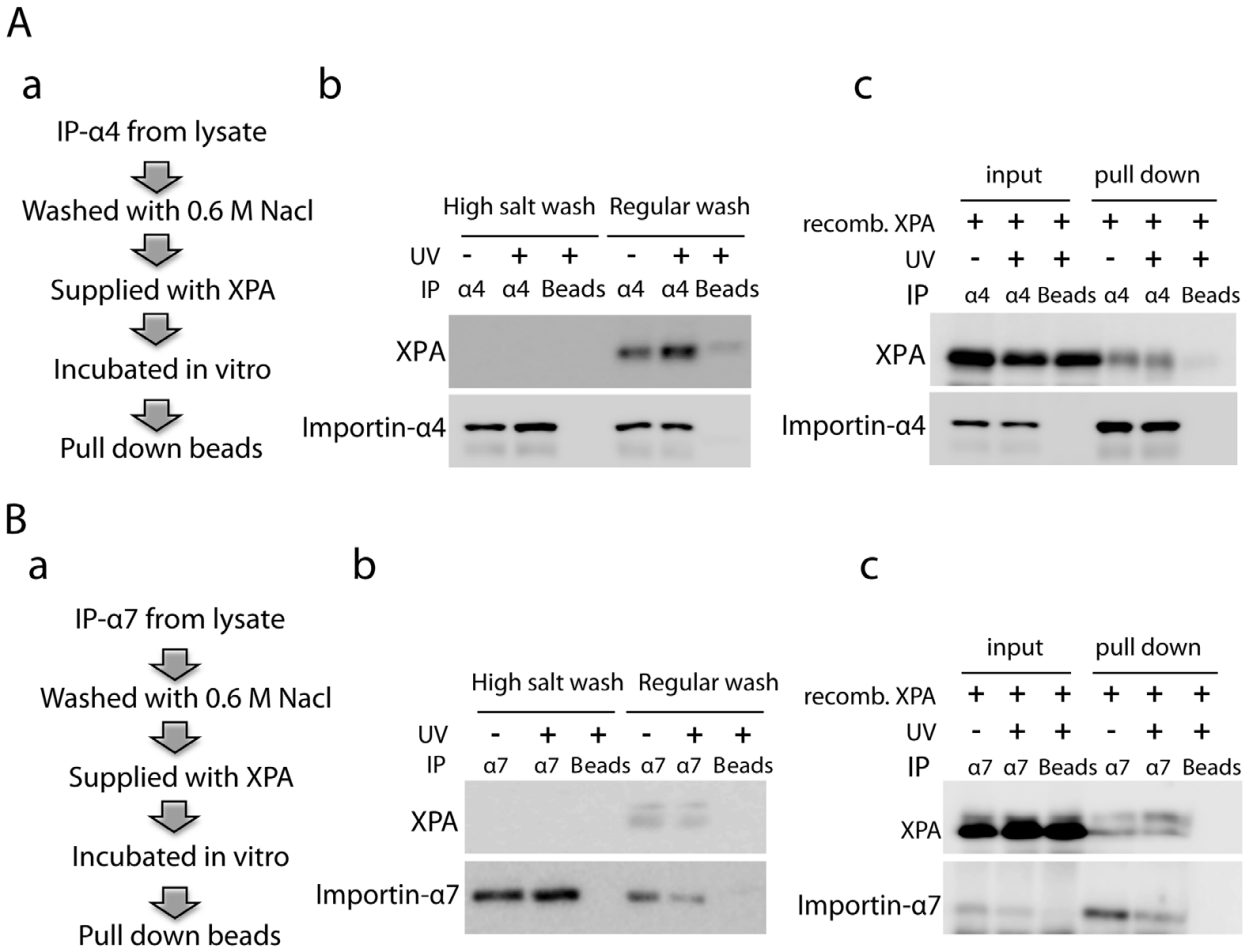
Demonstration of a direct interaction of importin-α4 or importin-α7 with XPA *in vitro*. **A.** *In vitro* interaction of importin-α4 and XPA. (a) A diagrammatic illustration of experimental procedures. H1299 cells were mock or UV-C irradiated (20 J/m^2^) followed by a ½ h recovery. Importin-α4 was immunoprecipitated as in Figure 3A, and the precipitate rinsed with high salt (0.6 M NaCl) to remove XPA and other proteins associated with importin-α4. Recombinant XPA protein was incubated with the immunoprecipitated importin-α4 and the complexes associated with the beads isolated by centrifugation. (b) Western blot analysis indicates that the XPA associated with the immunoprecipitated importin-α4 (Regular wash) was efficiently removed by the 0.6 M NaCl (High salt wash). (c) The ability of added recombinant XPA to bind to the immunoprecipitated importin-α4 was demonstrated by Western blotting of the bead-associated protein complexes. The left three lanes represent the mixture of IPed importin α4+ recombinant XPA as input while the right 3 lanes are the isolated complexes of IPed importin α4-XPA bound to the beads (pull down). **B.** Similar to A, except that importin-α7 was immunoprecipitated, treated with a high salt wash before incubation with recombinant XPA.

In a reciprocal experiment, cell lysates of XPA-deficient GM04429 cells were supplied with recombinant XPA, incubated, and then the XPA was immunoprecipitated using anti-XPA antibody. As shown in Figure 3B, importin-α4 and importin-α7 were co-immunoprecipitated with XPA. In contrast, probing the same blot with importin-α1 and importin-α5 antibodies showed no bands in the Western blotting (Figure 3B), indicating the preferred interactions of XPA with importin-α4 and importin-α7. Interestingly, the *in vitro* binding of recombinant XPA to importin-α4 in cell lysates did not increase when the cells used to prepare the lysates were exposed to UV irradiation (Figure 3B and Figure 4A–c). This observation is different from the results in Figure 3A where endogenous proteins were co-immunoprecipitated directly from cell lysates. This difference suggests that the binding affinity of XPA to importin-α4 itself is not affected by UV-irradiation of cells, but the binding is regulated in a UV-dependent manner, probably through the association with other cytoplasmic factors that may mask the XPA NLS [48] [49]. Indeed, it was previously reported that the UV-induced nuclear import of XPA was ATR-dependent [24]. Consistent with the previous observations, a reduction in the amount of XPA interacting with importin-α4 was observed in lysates produced from cells in which ATR had been knocked down by siRNA (Figure 3C).

To further determine whether importin-α4 and importin-α7 directly or indirectly interacted with XPA, *in vitro* protein-protein interaction experiments were performed. As illustrated in Figures 4A-a and 4B-a importin-α4 or importin-α7 was isolated by immunoprecipitation from cell lysates. Panel b of Figures 4A and 4B demonstrated that associated cellular XPA was released from the immunoprecipitated importin-α4 or importin-α7, respectively, by rinses with a high salt buffer which washes away interacting proteins as described previously [25], [36]. Then, purified recombinant XPA protein was added to the washed precipitates of importin-α4 or importin-α7, followed by incubation and a further wash with regular binding buffer. The pull down of recombinant XPA with importin-α4 and importin-α7 antibodies (Figures 4A–c and 4B–c) demonstrated that the interaction of importin-α4 or importin-α7 with XPA was indeed direct. Given the UV-dependent interaction of endogenous XPA and importin-α4 in cells, this result further suggests that the endogenous importin-α4-XPA interaction may be regulated by other protein factors in a UV irradiation-dependent manner. These regulatory factors are absent from the high salt-washed importin-α4- or importin-α7-beads.

### Knockdown of XAB1, the Proposed GTPase, did not Affect UV-induced XPA Import

**Figure 5:**
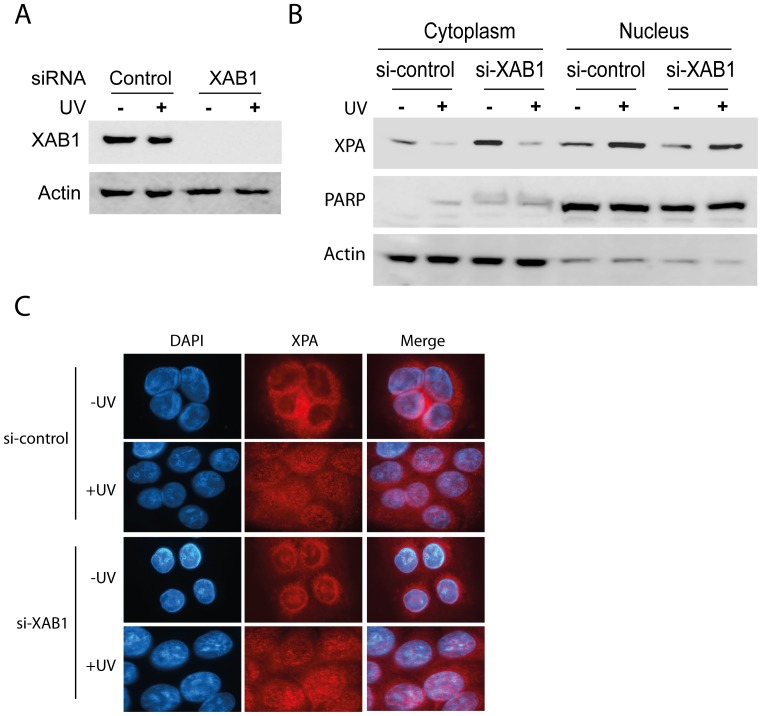
Knockdown of XAB1 did not reduce the UV-induced XPA nuclear import. **A.** western blotting confirmed siRNA knockdown efficiency of XAB1. A549 cells were transiently transfected with siRNA targeting XAB1. 72 hours post-transfection cells were mock treated or exposed to UV-C radiation (20 J/m^2^) followed by a 2-hr recovery. Whole cell lysates were prepared for western blotting analysis of XAB1. **B and C.** Demonstration that XAB1 was not needed for the nuclear localization of XPA in A549 cells. **B.** 72 hours post-transfection of control siRNA or siRNA targeting XAB1, cells were mock or 20 J/m^2^ of UV-C irradiated and allowed to recover for 2 hrs. Cytoplasmic and nuclear lysates were separated and loaded onto SDS-PAGE for analysis of XPA by western blotting. **C.** Immunofluorescence detection of XPA in the cells treated by UV-irradiation as in B.

Transportation of cargo protein in importin-α/−β protein complexes through the NPC requires energy derived from the activities of GTPases [40], [45] (Figure 2A). In a yeast two-hybrid system XAB1 was observed to bind XPA and it has been proposed to be the GTPase involved in XPA nuclear import [50]. To determine whether XAB1 protein is involved in human XPA nuclear transport, XAB1 was silenced by siRNA knockdown in cells (Figure 5A), followed by subcellular fractionation and analysis by Western blotting (Figure 5B) or by immunofluorescence detection of XPA (Figure 5C). Surprisingly, XAB1 knockdown had no effect on XPA nuclear import as compared with the control siRNA, suggesting that XAB1 alone was dispensable for the UV-induced nuclear import of cytoplasmic XPA in human cells.

## Discussion

**Figure 6:**
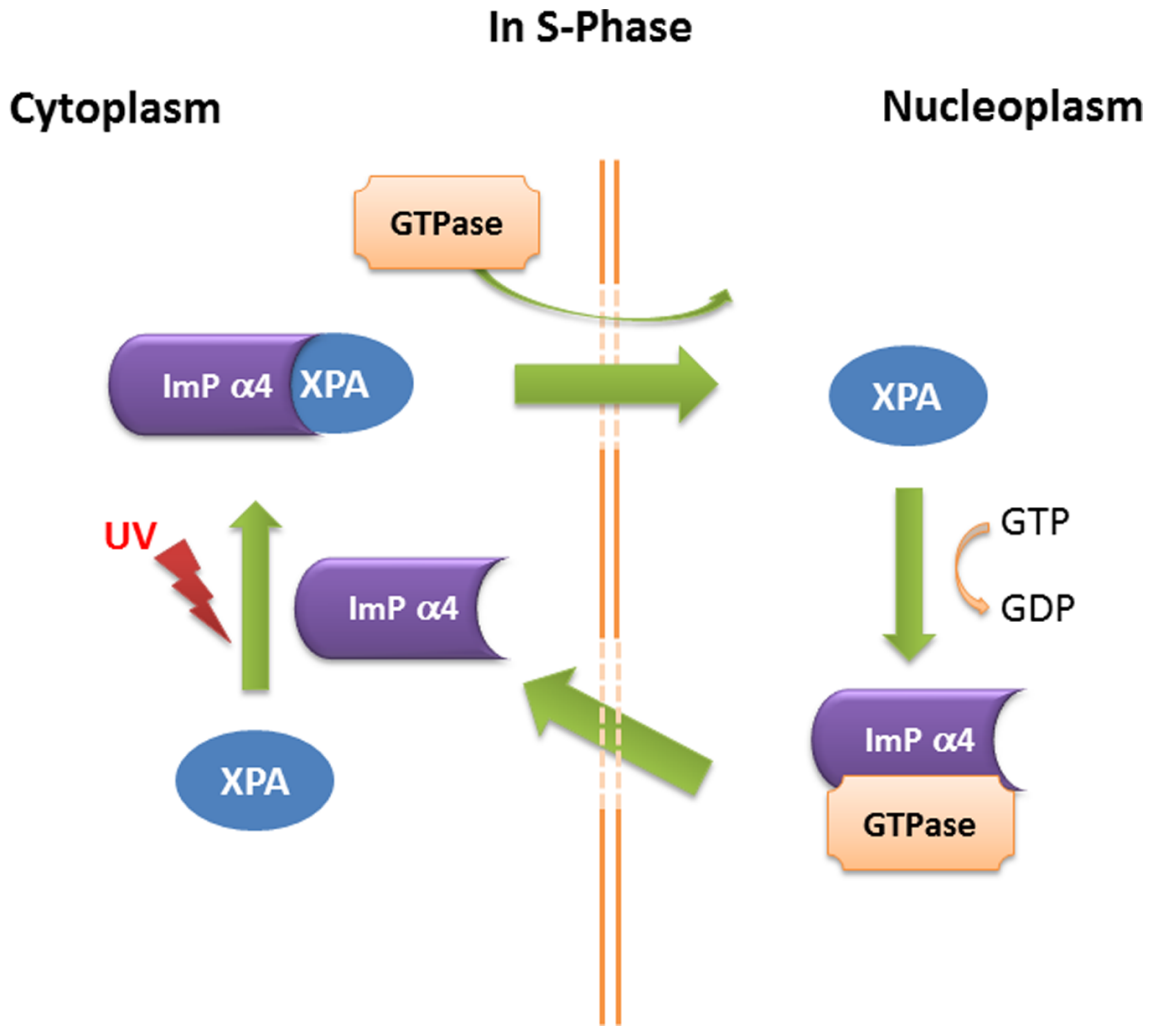
Proposed mechanism for the UV-induced nuclear import of XPA. XPA nuclear import is specific for the S phase of the cell cycle, thus this mechanism is described for the S phase only. In response to UV-C irradiation XPA forms a complex with importin-α4 (Imp α4) and importin-β (not shown) for nuclear import in a process facilitated by a GTPase other than XAB1.

XPA has crucial functions in NER. It is essential to understand the mechanism by which XPA is imported into the nucleus in response to DNA damage as NER depends on XPA functioning within the nucleus. In this study we identified the proteins involved in the nuclear import of XPA. We found that the DNA damage-induced nuclear import of XPA depended on an intact N-terminal NLS within the protein. In addition, importin-α4 and importin-α7 proteins were involved in the nuclear import of XPA. We demonstrated that importin-α4 and importin-α7 proteins directly interacted with XPA, but in cells, the interaction of XPA with importin-α4 was UV-induced and dependent on ATR. In contrast, the XAB1 protein, which has been proposed to be the GTPase involved in XPA nuclear import [50] was not required for the DNA damage-induced nuclear import of XPA. We conclude that importin-α4 is the transport adaptor for the UV-induced XPA nuclear import (Figure 6).

The observation that the nuclear import of XPA depended on its NLS in cells excludes the possibility that XPA was co-imported with other proteins containing a NLS, or imported by “alternative import mechanisms” [31]. These data also indicate that the same NLS was utilized by different adaptors, importin-α4 and/or importin-α7, for transporting XPA through the NPC. Interestingly, the binding of importin-α4 was largely induced by UV-irradiation within ½ hour after exposure (Figure 3A, left panel). This suggests that the efficient nuclear import of XPA may occur as early as ½ hour post UV-irradiation, consistent with our previous report [24]. In contrast, substantial XPA-importin-α7 interaction was observed in the absence of DNA damage and, thus, the DNA damage appeared to have no effect on the interaction (Figure 3A, right panel). These results indicate that although both importin-α4 and/or importin-α7 were required for XPA nuclear import, the requirement of importin-α4 was DNA damage-dependent while that of importin-α7 was not. Given that a small portion of XPA was present in the nucleus even without DNA damage, a possible scenario is that importin-α7 could be involved in the nuclear import of XPA independent of DNA damage (Figure S1D), while importin-α4 participated in the damage-dependent import of XPA.

In addition, it has been shown previously that, the DNA damage-induced nuclear import of XPA was cell cycle dependent, primarily occurring in S phase [33]. In G2-phase cells, XPA was localized to the nucleus regardless of DNA damage [33]. Thus, it is possible that importin-α7 is mainly responsible for nuclear import of XPA in G2 phase, while importin-α4 for S phase. This also may provide a possible explanation for the observation that knockdown of importin-α7 had less effect on the XPA nuclear import because the percentage of G2 cells in an unsynchronized cell population is only about 15–20%.

It previously was reported that the DNA damage checkpoint protein kinase ATR was involved in the regulation of the UV-induced XPA nuclear import^23^. Interestingly, here we showed that the UV-induced binding of importin-α4 to XPA was also ATR dependent (Figure 3C). Although ATR phosphorylates XPA at Ser196 in cells in responses to UV damage [26], we found that abolishing the phosphorylation of XPA had no effect on XPA nuclear import [25]. This suggests that the dependence of the XPA-importin-α4 binding on ATR may occur via ATR regulation of other cytoplasmic factors involved in regulating the XPA-importin-α4 binding [24], [33].

The NLS of XPA could not be efficiently recognized by importin-α4 in the absence of UV-irradiation (Figure 3A), likely due to the masking of the XPA NLS by the binding of other cytoplasmic factors [48], [49]. This hypothesis is validated by the observation that importin-α4 immunoprecipitated from both UV- and mock-treated cell lysates could bind recombinant XPA protein (Figure 4A, panel c). This ability of isolated and washed importin-α4 to bind recombinant XPA was observed for both H1299 lung carcinoma and GM04429 transformed fibroblast cells (Figures 3B and 4A), and suggests that this behavior is a general feature of the XPA-importin-α4 interaction. Elucidation of the details as to which cytoplasmic factors might mask the NLS and thus regulate this interaction, though beyond the scope of this study, merits future investigation. Finally, the protein XAB1 is a GTP-binding protein that was identified as an interacting partner with XPA in a yeast two-hybrid system, and was proposed to be the GTPase involved in XPA nuclear import [50]. However, our result show that siRNA knockdown of XAB1 had no effect on the UV-induced nuclear import of XPA (Figure 5). This inconsistency could be due to the different environments within human cells and the yeast model system. Our data support a GTPase other than XAB1 as being involved in the nuclear import of XPA in human cells.

Also, it is worth noting the reports of immunofluorescence microscopic analyses that showed that XPA was fully localized to the nucleus even in the absence of DNA damage [38], [51], [52]. Given that these reports exclusively focused on images of individual cells transfected with XPA-expression constructs with or without GFP tags, the observations may not be inconsistent with the results of this study since XPA subcellular localization in unstressed cells is cell cycle-dependent [33]. Indeed we previously reported that XPA was predominantly localized to the nucleus in unstressed G2-phase cells [33]. Though immunofluorescence images may accurately depict XPA distribution within an individual cell they do not reflect the subcellular localization of XPA within a population of cells as do our biochemical fractionation studies which represent the average XPA distribution within millions of cells [24], [32], [33]. Also, in general G_2_ phase cells present the sharpest images of XPA location since the protein appears more concentrated within the smaller, defined nuclear structure. In addition, we recently found that the same monoclonal anti-XPA antibody yields distinctly different XPA distribution patterns depending on the method of cell fixation. XPA is predominately located in the cytoplasm of undamaged cells fixed with methanol; in contrast, formaldehyde fixation resulted in a predominately nuclear localization in undamaged cells (data not shown). Apparently, the fixation method affects the antigen display with methanol revealing the same antigens as seen in the western blots of SDS-denatured proteins. Formaldehyde rather than methanol was employed in the reports of a nucleus-only location for XPA [38], [51], [52]. Even the basal level of nuclear XPA in the fractionation assays may vary somewhat between experiments, likely due to culture variations affecting the proportion of cells in the G_2_ phase [32], [33]. However, we observed a 2.8-fold average increase in the XPA and importin-α4 interaction after UV irradiation in at least three independent experiments. Finally it is unknown if the GFP tag could have any effects on XPA in cytoplasm.

Due to its indispensable role in human NER, including both global genome and transcription-coupled NER sub-pathways, XPA may serve as a potential target for sensitization to cancer chemotherapy (e.g. cisplatin) via manipulation of available nuclear XPA, either at the transcriptional or post-transcriptional level [53], [54], [55]. Indeed, we previously showed that cisplatin, like UV irradiation, induced XPA nuclear import [24], [32]. Furthermore, recent studies indicate that targeting protein-trafficking pathways altered the sensitivity of melanoma cells to chemotherapy [34]. The results from this study suggest that targeted disruption of the XPA-importin-α4 complex could be a potential strategy to reduce the nuclear level of XPA given that NER occurs exclusively in the nucleus. Since the DNA damage-induced nuclear import of XPA occurs primarily in S-phase [33], the inhibition is expected to be specific to replicative cells, typically cancer cells, without interruption of XPA functions in cells in other cell cycle phases.

## Supporting Information

Figure S1 **Requirement of NLS for XPA nuclear import in different types of cells.** **A-C.** The same XPA constructs as in Figure 1B were transit transfected into human 293T and GM02249 cells, as well as human primary fibroblasts BJ cells. Cells were mock or 20 J/m^2^ of UV-C irradiated and allowed to recover for 2 hrs. Fractionation and Western blotting assessed the subcellular localizations of XPA molecules. **D.** Effects of siRNA knockdown of importin α proteins on XPA nuclear import in the absence of UV. H1299 cells were transfected with indicated siRNAs. At 48-hours post transfection, cellular fractions (C for cytoplasm, N for nucleus) were collected and Western blotting was employed to assess the subcellular localizations of XPA.
(TIF)
