# Supplementary figures and images for: UV-Induced Nuclear Import of XPA Is Mediated by Importin-α4 in An ATR-Dependent Manner
Source: PLoS One. 2013 Jul 8;8(7):e68297. doi: 10.1371/journal.pone.0068297 (PMC3704644; doi:10.1371/journal.pone.0068297)

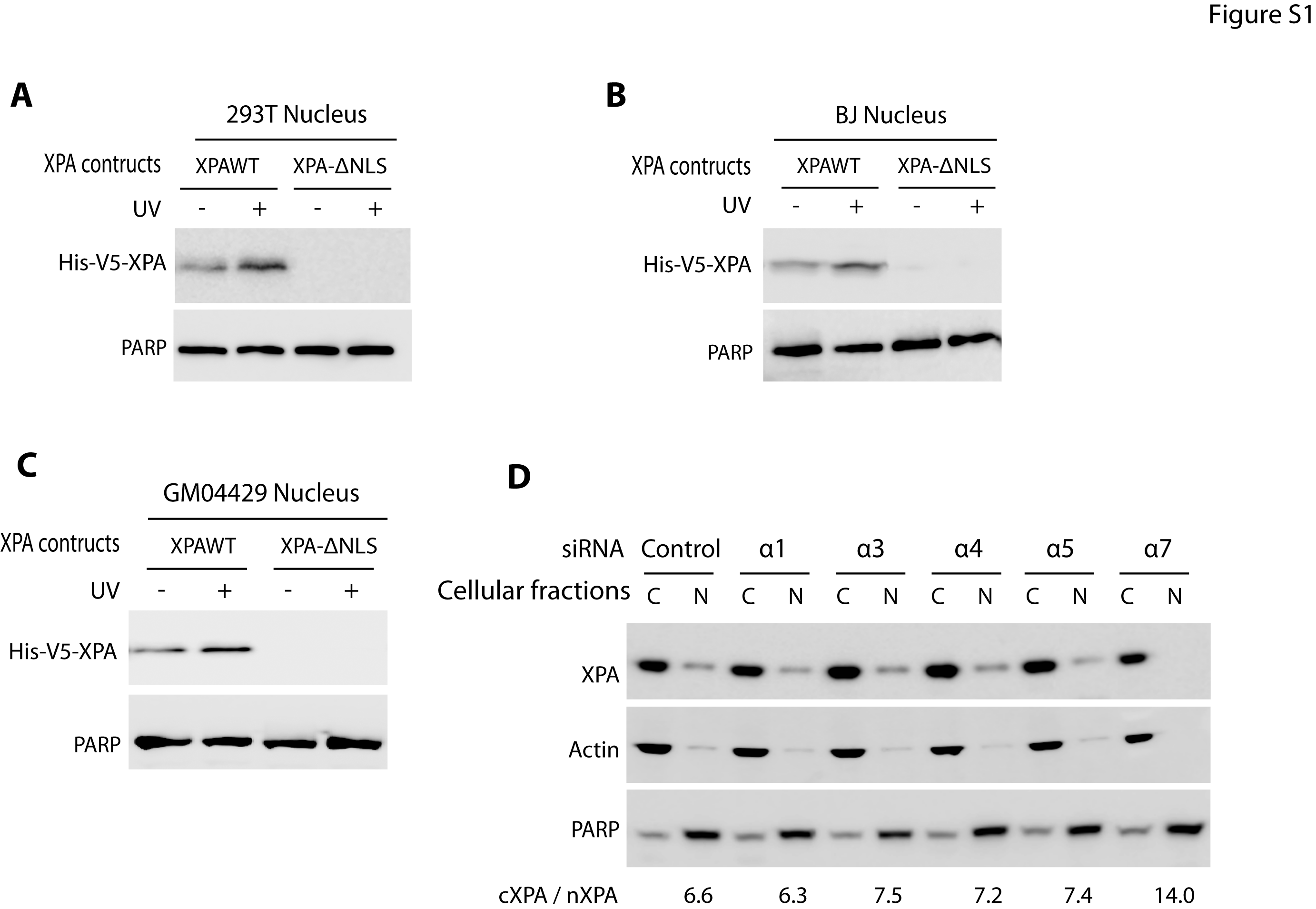

Supplement: Figure S1 — Requirement of NLS for XPA nuclear import in different types of cells. A-C. The same XPA constructs as in Figure 1B were transit transfected into human 293T and GM02249 cells, as well as human primary fibroblasts BJ cells. Cells were mock or 20 J/m2 of UV-C irradiated and allowed to recover for 2 hrs. Fractionation and Western blotting assessed the subcellular localizations of XPA molecules. D. Effects of siRNA knockdown of importin α proteins on XPA nuclear import in the absence of UV. H1299 cells were transfected with indicated siRNAs. At 48-hours post transfection, cellular fractions (C for cytoplasm, N for nucleus) were collected and Western blotting was employed to assess the subcellular localizations of XPA. (TIF) [file pone.0068297.s001.tif]
